# Dietary Protected Butyrate Supplementation of Broilers Modulates Intestinal Tight Junction Proteins and Stimulates Endogenous Production of Short Chain Fatty Acids in the Caecum

**DOI:** 10.3390/ani12151940

**Published:** 2022-07-30

**Authors:** Gábor Mátis, Máté Mackei, Bart Boomsma, Hedvig Fébel, Katarzyna Nadolna, Łukasz Szymański, Joan E. Edwards, Zsuzsanna Neogrády, Krzysztof Kozłowski

**Affiliations:** 1 Division of Biochemistry, Department of Physiology and Biochemistry, University of Veterinary Medicine, István utca 2, H-1078 Budapest, Hungary; matis.gabor@univet.hu (G.M.); mackei.mate@univet.hu (M.M.); neogrady.zsuzsanna@univet.hu (Z.N.); 2 Palital Feed Additives B.V., De Tweede Geerden, 5334 LH Velddriel, The Netherlands; b.j.boomsma@gmail.com (B.B.); j.edwards@palital.com (J.E.E.); 3 Nutrition Physiology Research Group, Institute of Physiology and Nutrition, Hungarian University of Agriculture and Life Sciences, Gesztenyés Str. 1, H-2053 Herceghalom, Hungary; hullarne.febel.hedvig@uni-mate.hu; 4 Department of Poultry Science and Apiculture, Faculty of Animal Bioengineering, University of Warmia and Mazury, Oczapowskiego 5, 10-719 Olsztyn, Poland; katarzyna.nadolna@uwm.edu.pl (K.N.); lukasz.szymanski@uwm.edu.pl (Ł.S.)

**Keywords:** butyric acid, organic acids, poultry, chicken, growth performance, gut, epithelium, microbiota

## Abstract

**Simple Summary:**

The aim of this study was to evaluate the effect of protected butyrate supplementation in broiler nutrition. Two experiments were conducted: (i) a performance study (1–35 days of age) and (ii) a study on the gut integrity and microbiome (1–21 days of age). The growth performance of birds, abundance of intestinal tight junction proteins, the quantitative characteristics of caecal microbiome and intestinal short chain fatty acid (SCFA) concentrations were investigated in these studies. Based on the results, it could be concluded that the addition of protected butyrate to broiler diets improved growth performance and intestinal integrity in accordance with the modulation of the caecal microbiome and the stimulated endogenous production of SCFA in the caecum. These findings provide important information that increases the understanding of the complex effects of the biologically most active SCFA, butyrate, on the overall health status and metabolism of the chicken.

**Abstract:**

Short chain fatty acid (SCFA) butyrate has various beneficial effects on the gut microbiota as well as on the overall health status and metabolism of the host organism. The modulatory role of butyrate on gut barrier integrity reflected by tight junction protein expression has been already described in mammalian species. However, there is limited information available regarding chickens. Therefore, the main aim of this study was to monitor the effects of protected butyrate on claudin barrier protein and monocarboxylate transporter 1 abundance in different gastrointestinal segments of chickens as well as the growth performance of broiler chickens. The effect of protected butyrate on the caecal microbiota was monitored by quantifying the concentrations of total eubacteria and key enzymes of butyrate production. Furthermore, intestinal SCFA concentrations were also measured. Based on the data obtained, protected butyrate increased the overall performance as well as the barrier integrity of various gut segments. Protected butyrate also positively affected the SCFA concentration and composition. These findings provide valuable insight into the complex effects of protected butyrate on broiler gut health, highlighting the beneficial effects in improving intestinal barrier integrity and performance parameters.

## 1. Introduction

In monogastric species, the short chain fatty acid (SCFA) butyrate is mainly produced in the gut from the caecal microbial fermentation of structural polysaccharides derived from dietary fibers [1]. In chicken caeca, Bacteroidetes and Firmicutes are the major bacterial phyla involved in the breakdown of non-starch polysaccharides (NSPs) and bypass oligosaccharides to the constituting monosaccharides, which are degraded via the glycolytic or pentose phosphate pathway to acetyl-CoA [2]. Subsequently, two acetyl-CoA molecules can be condensed to butyryl-CoA, which is then transformed to butyrate via pathways involving one of two key enzymes: butyrate kinase or butyryl-CoA acetate CoA transferase (BCACT) [3].

Endogenously synthesized butyrate plays a key role in maintaining intestinal health via multiple mechanisms. Butyrate improves the balance of the gut microbiota [4], enhances intestinal barrier integrity, influences intestinal epithelial cell proliferation, regulates epithelial inflammation through production of anti-inflammatory cytokines [5], promotes the enteric immune system and decreases oxidative stress [6,7,8]. As well as caecal SCFA production, orally applied butyrate is widely used as a feed additive to improve health and growth performance of broiler chickens [9]. However, orally administered non-protected butyrate salts are rapidly absorbed from the acidic proximal sections of the gastrointestinal tract. As such, protected forms of butyrate (e.g., microencapsulated butyrate and various esters) are used to deliver butyrate more distally towards the small intestines [10,11].

The wide range of beneficial effects of butyrate is based on its numerous epigenetic and receptor-mediated effects in the gut and the extraintestinal tissues [1]. Butyrate can highly affect gene expression by causing histone hyperacetylation [12], and it is likely to be a potent modulator of insulin homeostasis in broilers, selectively upregulating insulin receptor β (IR β), possibly contributing to increased insulin sensitivity [13,14].

Concerning the intestinal effects of butyrate, it was reported that butyrate could increase gut barrier integrity by influencing the expression and distribution of tight junction proteins, such as claudins and occludin. For instance, butyrate selectively upregulated claudin-3 and -4 on both mRNA and protein level in porcine epithelial IPEC-J2 cell cultures [15]. In another in vitro study with IEC-6 rat-derived enterocyte monolayers, sodium butyrate enhanced the gene expression of claudin-1 via facilitating the interaction between the transcription factor SP1 and the promoter of the claudin-1 gene [16]. A limited number of studies with chickens have also provided evidence of similar effects occurring in vivo [17,18]. Use of microencapsulated butyrate as feed supplement alleviated gut barrier injuries caused by necrotic enteritis (*Clostridium perfringens* infection), which was consistent with enhanced claudin-1, -4 and occludin gene expression in the jejunal mucosa of the broilers. The stimulatory action of inulin supplementation on claudin-1 mRNA abundance was also confirmed in the ileum of broiler chickens and was suggested to be in connection with the inulin-mediated increase in microbial butyrate production [18].

As indicated by the aforementioned studies, whilst the modulatory role of butyrate on tight junction protein expression pattern is already described, there is currently limited in vivo information available about the chicken. Hence, the main goal of the present study was to monitor the alterations of claudin-1 and -3 protein levels in the mucosa along the digestive tract of chickens fed with diets containing protected butyrate. Claudin-1 and -3 were selected to study since they are considered as the major barrier-forming claudins, and their presence was already confirmed in the intestines of poultry [19]. Furthermore, the abundance of monocarboxylate transporter 1 (MCT- 1) as the main SCFA transporter was also assessed in the gut segments as it can strongly influence butyrate absorption and consequently butyrate’s extraintestinal actions. The effect of protected butyrate on the caecal microbiota was monitored as well by targeting 16S rRNA reflecting total eubacteria count, and key enzymes of butyrate production (i.e., butyrate kinase and BCACT). Intestinal SCFA concentrations were also monitored, and a separate performance study was carried out to confirm the effect of protected butyrate’s action on growth performance. These investigations together provide insight into the complex effects of orally supplemented protected butyrate on gut health, highlighting the putative role of protected butyrate in improving intestinal barrier integrity and contributing to improved performance parameters.

## 2. Materials and Methods

The research reported in this paper was performed in two parts: a performance study (Section 2.1) and a gut mechanism study (Section 2.2). The broiler performance study was conducted with a growth and a fattening period that spanned 1–35 days of age of the birds. The gut mechanism study was conducted with broilers during the most intensive growing period, i.e., from 1–21 days of age. 

### 2.1. Performance Study

#### 2.1.1. Animals and Housing

Healthy male day-old commercial Ross 308 broiler chicks (average of 37.4 g body weight) were distributed at random into 40 pens (0.7 m × 1.0 m) of 9 birds/pen (14 birds/m^2^). Pens had a concrete floor and contained used litter from a previous study. The programmable artificial lighting of the broiler house was set at 24 h of light for the first 2 days of the study, followed by 18 h light and 6 h of dark per day until the end of a 35-day study. Environmental conditions during the study (temperature and ventilation rate) were automatically controlled and appropriate for the age of the broilers. Diets were provided to the broilers ad libitum from feeders. Water was provided ad libitum from nipple drinkers. The study terminated after 35 days, and birds were delivered to the slaughterhouse. All procedures in the performance study were performed in accordance with the principles of the European Union and Polish Law on Animal Protection in compliance with current quality standards for EU feed additive applications. The performance study was conducted by qualified veterinarians who performed all procedures that involved the handling of the birds. No action involving pain or suffering was practiced. This performance study was run in accordance with Directive No. 2010/63/EU and did not require the approval of the Local Ethics Committee based on the regulation of the Ethic Committee of November 2019 (resolution No. 174/2019).

#### 2.1.2. Experimental Design and Diets

During the 35-day study period, the broilers received dietary treatments with 10 pens allocated per treatment (i.e., 90 broilers). The treatments were different dosage levels of a fat matrix encapsulated blend of sodium and calcium butyrate (70% butyrate; Intest-Plus Quattro^®^, Palital Feed Additives B.V., Velddriel, the Netherlands), hereinafter referred to as protected butyrate. The dosages were: Control (0 g of protected butyrate/metric ton (T) of starter & grower diets), Low (300 g of protected butyrate/T starter & grower diets), Medium (600 g of protected butyrate/T starter diet and 300 g of protected butyrate/T grower diet) and High (1000 g of protected butyrate/T starter diet and 300 g of protected butyrate/T grower diet).

A single batch for each of the starter (1–14 days) and grower (15–35 days) basal diets (Table 1) was prepared and split to prepare the feed for each of the dietary treatments. Basal diets were calculated to meet or exceed the nutrient requirements recommended for Ross 308 broilers. 

#### 2.1.3. Measurements

On a pen basis, body weight of the broilers (day 1, 14, and 35 of the study) and feed consumption (day 14, and 35 of the study) were measured throughout the study. Based on these results, the average daily gain, average daily feed intake and feed conversion ratio were then calculated.

#### 2.1.4. Statistical Analysis

The Statistical software package version 13.1 [20] was used to determine whether variables differed between treatment groups. The one-way ANOVA and Tukey’s Test for Post-Hoc Analysis were used to compare the means. Polynomial Regression was used to study the linear and quadratic relationship. The results are presented in the tables as mean values with pooled standard errors. Data were checked for normal distribution before the statistical analysis was performed. Differences were considered to be significant at *p* ≤ 0.05.

### 2.2. Gut Mechanism Study

#### 2.2.1. Animals and Housing

Forty healthy male day-old commercial Ross 308 broiler chicks (average of 46.0 g body weight) were distributed at random into two pens with 20 birds/pen (14 birds/m^2^). The floor pens were bedded with chopped wheat straw. Environmental and climatic conditions were set according to the requirements of the Ross technology [21]. Diets and water were provided to the broilers ad libitum. Housing and treatment of the chickens were carried out in strict accordance with the national and international laws as well as with the institutional guidelines at the University of Budapest. Experimental procedures were approved by the Government Office of Pest County, Food Chain Safety, Plant 4 Protection and Soil Conservation Directorate, Budapest, Hungary (number of permission: PEI/001/1430-4/2015).

#### 2.2.2. Experimental Design and Diets

Each of the two pens was allocated to a dietary treatment: control (no butyrate supplementation, i.e., only basal feed) and protected butyrate (1000 g /T basal feed). As indicated above (Section 2.1.2), the protected butyrate was a fat matrix encapsulated blend of sodium and calcium butyrate (Intest-Plus Quattro®, Palital Feed Additives B.V., Velddriel, the Netherlands). Unlike in the performance study, the protected butyrate dosage level was kept the same in both the starter and grower diets in order not to confound the analysis performed in this study relative to bird age. Details of the basal diets for the starter (1–10 days of age) and grower (11–21 days of age) phases are provided in Table 2.

#### 2.2.3. Samplings

At two different time points (day 10 and day 21), ten chickens from each group were slaughtered without prior starvation using carbon dioxide narcosis (i.e., 10 birds/group/time point). Ingesta samples were taken from the following sections of the gastrointestinal tract (GIT): proventriculus, gizzard, duodenum, jejunum, ileum and caeca. As the proventriculus and gizzard should be functionally considered as one compartment, their ingesta was mixed as one sample. As the sample was mostly derived from the gizzard ingesta, it is referred to as simply the “gizzard”. With regard to the duodenum, samples were taken from the whole duodenal loop (content of the descending and ascending duodenum was mixed). Concerning the jejunum and the ileum, ingesta was taken from the intestinal section starting 10 cm proximally from the Meckel’s diverticulum and finishing 10 cm distally from it and mixed to make a combined sample, which is referred to as jejunum+ileum. Caecal content was taken as a mixture of ingesta from both the left and right sacs. All ingesta samples were then split into two aliquots and then immediately shock-frozen in dry ice before being stored at −80 °C until further processing.

After ingesta sampling, the appropriate gut sections were longitudinally opened, flushed with chilled physiological saline solution. For each section, the mucosa was then gently scraped with a microscope slide. In the case of the stomachs, the mucosa of the proventriculus and gizzard was collected. As with the ingesta, these mucosa samples were combined and referred to simply as “gizzard”. Regarding the mucosa samplings from the duodenum, jejunum+ileum and caeca, the same locations were used as for the ingesta collection. All the mucosa samples were immediately shock-frozen in dry ice before being stored at −80 °C until further processing.

#### 2.2.4. Assessment of Claudin-1, Claudin-3 and MCT-1 Protein Abundance

After thawing on ice, mucosa samples were homogenized in M-PER lysis buffer (adding approx. 1 mL to 100 mg tissue) freshly supplemented with 1% Halt Protease Inhibitor Cocktail (both from Thermo Fisher Scientific, Waltham, MA, USA) with a Dounce homogenizer (Thermo Fisher Scientific, Waltham, MA, USA). Homogenates were centrifuged at 5000× *g* for 10 min at 4 °C, and the supernatant was used for further analyses.

Total protein concentration of the mucosa homogenates was assayed using the Pierce^TM^ Bicinchoninic Acid (BCA) Protein Assay (Thermo Fisher Scientific, Waltham, MA, USA) applying bovine serum albumin (BSA) as the standard and adding 25 μL sample to 200 μL reagent mixture (50:1 mixture of reagents A and B, respectively). Absorbance was measured after 30 min incubation at 37 °C at 562 nm using a Multiskan GO 3.2 reader (Thermo Fisher Scientific, Waltham, MA, USA).

Protein abundance of claudin-1, claudin-3 and MCT-1 within the samples was measured with chicken specific quantitative sandwich enzyme-linked immunosorbent assay (ELISA) kits (cat. No MBS026451, MBS085865, MBS9392435 for claudin-1, claudin-3 and MCT-1, respectively) as described by the manufacturer (MyBioSource, San Diego, CA, USA). Briefly, 50 μL sample and 100 μL HRP-Conjugate Reagent were pipetted into every well of a 96-well microtiter plate and incubated for 60 min at 37 °C. After 4 times washing with Washing Buffer, 50 μL of Chromogen Solution A and 50 μL Chromogen solution B were added to each well. Plates were gently shaken and then incubated for 15 min at 37 °C. After this incubation, the reaction was blocked by adding 50 μL Stop Solution, and six absorbance values were measured at 450 nm within 15 min using a Multiskan GO 3.2 reader. Protein abundance values were standardized to total protein concentrations in order to ensure reliable comparisons.

#### 2.2.5. Measurement of SCFA Concentrations of Ingesta

The SCFA of ingesta was extracted and then analyzed using gas chromatography. An extraction was required in order to ensure detection of all the SCFA, including any butyric acid that had not yet been released from the protected butyrate. For extraction, ingesta samples (800 mg) were mixed with the following: 8 mL of distilled water, 135 μL of NaOH solution (0.2 M), 10 mL of chloroform and 700 μL of isovaleric acid solution (200 mg/100 mL) as an internal standard. The sample was vortexed (600 rpm 10 min at room temperature), then the layers were separated with centrifugation (3500 rpm) for 5 min at room temperature. Thereafter, 2100 μL of the water phase was mixed with 250 μL metaphosphoric acid (28.5 *w*/*w*%).

For the analyses, a GCMS-QP2010 SE Shimadzu Gas Chromatograph was used. The capillary column used was a Zebron ZB-WAXplus type (30 m × 0.25-mm id, and 0.25-μm film thickness; Phenomenex, Torrance, CA, USA). The GC conditions were as follows: the column temperature was programmed from 75 °C to 175 °C with an increase of 11 °C/min, and finally held for 1 min. Injection mode was splitless and sampling time was 0.7 min. Flow control mode was linear velocity (49.4 cm/sec), and the pressure was 119.9 kPa. Total flow was 77.1 mL/min, column flow: 1.81 mL/min, and purge flow was 3 mL/min. The ion source and interface temperatures were both 200 °C.

Concentration (nmol/g) of each SCFA in the sample was calculated using the following equation = integral value of SCFA × weight of internal standard in the sample (mg)/integral value of internal standard/relative sensitivity of SCFA × 1,000,000/weight of ingesta sample (g)/molecular weight of SCFA. Relative sensitivity or response factor of each SCFA was determined before analysis of SCFA content in ingesta samples by using the following equation: 

Response Factor Equation = integral value of each calibration standard of SCFA × concentration of internal standard/integral value of internal standard × concentration of each calibration standard of SCFA.

#### 2.2.6. Quantitative PCR Analysis of Targeted Genes in Caecal Ingesta

DNA was extracted from caecum ingesta samples in order to enable the quantitative analysis of total eubacteria and genes encoding for butyrate kinase and BCACT enzymes with quantitative PCR.

##### DNA Extraction

Bacterial DNA was extracted from the caecum ingesta samples as previously described [22] with some minor modifications. Initially, caecum ingesta samples (0.2–0.5 g) were suspended in 5 mL of phosphate buffered saline with EDTA, and then vortexed vigorously for 5 min. Solid particles of the suspension were then sedimented on ice for 20 min. One ml from each sample was transferred to a clean microcentrifuge tube and subjected to centrifugation at 18,000× *g* for 10 min in order to pellet the bacterial cells. Subsequently, the pellet was re-suspended in 600 µL of lysis buffer containing EDTA and Tris-HCl with 20 µL of proteinase K. The suspension was transferred to a screw-cap microcentrifuge tube containing 0.4 g of sterile glass beads, and the suspensions were incubated at 65 °C for 60 min with vortexing for 30 s (1400 rpm) at 10 min intervals. The bacterial cells were disrupted by three 1-min rounds of bead beating (MP Biomedicals, Santa Ana, CA, USA) at 6.5 m/s. DNA was purified from the homogenates using phenol-chloroform-isoamyl alcohol (24:1) extraction at 10,000× *g* for 10 min, followed by chloroform-isoamyl alcohol purification at 10,000× *g* for 10 min. DNA was precipitated by addition of 0.6 volumes of 100% isopropanol, and pelleted by centrifugation at 18,000× *g* for 10 min. Finally, the DNA pellet was washed twice with 1 mL of ice cold 70% ethanol, dried and re-suspended in 100 µL of Tris-EDTA buffer.

##### Quantitative PCR Analysis

Quantitative PCR analyses were conducted with a 16S rRNA gene-targeted assay on total eubacteria and with eight individual assays targeting the genes encoding for butyrate kinase and BCACT enzymes (EpiHealth™ analysis panel, Alimetrics Diagnostics Ltd., Espoo, Finland) using SYBR Green I chemistry.

The amplifications were performed in a 96-well plate with an ABI Prism Sequence Detection System 7500 instrument (Thermo Fisher Scientific Inc., Waltham, MA, USA) in a volume of 15 µL with SYBR Select Master Mix (Thermo Fisher Scientific Inc., Waltham, MA, USA), 0.25 µM of both primers, and 5 µL of 1:100 diluted template DNA or deionized sterile water as a no-template control (NTC). The thermal cycling conditions used involved one cycle of preheating at 50 °C for 2 min and initial denaturation at 95 °C for 10 min followed by 40 cycles of denaturation at 95 °C for 15 s and primer annealing and extension at primer-specific annealing temperature for 60 s. To determine the specificity of the PCR reactions, a melting curve analysis was carried out in conjunction with each amplification run by slow cooling from 95 °C to 60 °C, with fluorescence collection at 0.3 °C intervals.

Tenfold serial dilutions ranging from 1 × 10^8^ to 1 × 10^2^ of synthetic target gene copies (gBlocks^®^ Gene Fragments, IDT, Coralville, IA, USA) were included on each 96-well plate. The fractional cycle number at which the fluorescence passed the threshold (set at 0.3 fluorescent units) was determined for the unknowns and compared with the standard curves. Taking into account the original quantity of starting material, elution volume and PCR template dilution, the numbers of target genes were determined per g of caecum ingesta.

#### 2.2.7. Statistical Analyses

Data were statistically analyzed with the R 3.2.2 software, by using multi-way ANOVA and Tukey’s Test for Post-Hoc Analysis after confirming normal distribution of the data. For the quantitative PCR data, analysis of the data was processed after Log10 transformation. Differences were considered to be significant at *p* ≤ 0.05. In the Results section, *p*-values belonging to the main effects (treatment, age) and to the interactions are presented.

## 3. Results

### 3.1. Performance Study

Protected butyrate significantly increased the body weight of the broilers at the end of both the starter (day 14) and grower (day 35) phases in a dose dependent manner (Table 3). For the starter phase, significantly increased body weights relative to the control were obtained with the medium and high dosage. For the grower phase, only the medium dosage significantly increased body weight relative to the control.

For the starter and grower phases, average daily gain was significantly increased by protected butyrate (Table 3). The dosage related effects in both phases were comparable to that for the body weight. With respect to the whole study period, the low and medium dosages resulted in significant increases in average daily gain relative to the control.

In contrast to the above, average daily feed intake was not significantly affected by treatment with protected butyrate (Table 3). Consistent with this, protected butyrate significantly improved the feed conversion ratio (FCR). Relative to the control, the FCR was significantly increased during the grower phase as well as over the whole study period in a dose dependent manner (Table 3). During the grower phase, both the medium and high dosages significantly decreased the FCR. With respect to the whole study period, all dosages significantly decreased the FCR with significant differences also between the low and high dosages.

The livability of the control group during the study was high, and protected butyrate had no impact on this parameter (Table 3).

### 3.2. Gut Mechanism Study

#### 3.2.1. Claudin-1, Claudin-3 and MCT-1 Protein Abundance

Both of the tight junction proteins, claudin-1 and claudin-3, as well as the SCFA transporter MCT-1 were significantly affected by age throughout the gut (Table 4). For each gut site, the abundance of these proteins in the mucosa was always significantly higher on day 10 relative to day 21.

Protected butyrate had a significant effect on claudin-1 protein abundance in all gut sites with the exception of the caecum (Table 4). Relative to the control, protected butyrate increased claudin-1 abundance in the gizzard and jejunum+ileum and decreased it in the duodenum. There was no significant age × treatment in any of the gut sites for claudin-1 protein abundance.

In contrast to claudin-1, protected butyrate only significantly affected claudin-3 protein abundance in the jejunum+ileum, which was increased relative to the control (Table 4). In the gizzard, there was a significant age × treatment interaction due to protected butyrate decreasing claudin-3 abundance on day 10 and increasing it on day 21.

MCT-1 protein abundance was significantly affected by protected butyrate in all gut sites except the caecum (Table 4). In the gizzard and jejunum+ileum, protected butyrate resulted in a significant increase in MCT-1 abundance. In contrast, in the duodenum, a significant decrease occurred that had a significant interaction with bird age due to a stronger decrease being evident on day 10 compared to day 21. Whilst no effect of protected butyrate occurred in the caecum, a significant interaction occurred between protected butyrate and bird age. A strong decrease in MCT-1 abundance in the caecum was evident on day 10, whereas on day 21 a small increase occurred. 

#### 3.2.2. SCFA Concentrations in Gut Digesta

Unlike with protein abundance, the effects of age on the concentration of SCFA varied with gut site and parameter (Table 5). Total SCFA concentration significantly decreased with the age in all gut sites with the exception of the duodenum, where a significant increase occurred instead. Age did not result in significant differences for any of the individual SCFA in the gizzard, unlike with the other gut sites. In the duodenum, the concentration of acetate and propionate both significantly increased with age. In the jejunum+ileum, acetate, propionate and butyrate concentrations all significantly decreased with age, whilst valerate was unaffected. In the caecum, acetate significantly decreased with age whilst propionate and valerate significantly increased and butyrate was unaffected.

Protected butyrate supplementation had an effect on SCFA in all gut sites. Butyrate concentration significantly increased in the gizzard, however, the acetate concentration significantly decreased to a greater extent. This resulted in a significant decrease in total SCFA. A significant age × treatment interaction also occurred in the gizzard in relation to propionate, which increased in concentration on day 10 but decreased on day 21.

In the duodenum, all SCFA parameters were affected by protected butyrate with the exception of valerate. Protected butyrate significantly decreased total SCFA concentration, despite a significant increase in butyrate concentration, due to a greater significant decrease in acetate. For both total SCFA and acetate, a significant age × treatment interaction was found due to a larger decrease on day 21 compared to day 10. Propionate concentration was also significantly affected by protected butyrate and had a significant age × treatment interaction, i.e., being significantly increased on day 10 and decreased on day 21.

In the jejunum+ileum, protected butyrate had a significant effect on butyrate concentration and a significant age × treatment interaction also occurred. Butyrate concentration was increased on day 10 to a greater extent relative to day 21. Valerate was significantly decreased by protected butyrate supplementation. A significant age × treatment interaction was found for propionate, with an increase on day 10 and a decrease on day 21.

In the caecum, the total SCFA was at least 10-fold higher than for the other gut sites, as expected due to the substantial microbial production of SCFA, and was significantly increased by protected butyrate. This was due a significant increase in all SCFA with the exception of valerate. The increase in butyrate concentration (day 10, +2.53 µmol/g digesta; day 21, +3.2 µmol/g digesta) in the caecum associated with protected butyrate supplementation was at least 10–100-fold higher than the increases seen in the other gut sites due to protected butyrate.

#### 3.2.3. Targeted Caecal Microbiota Analysis

**Figure 1:**
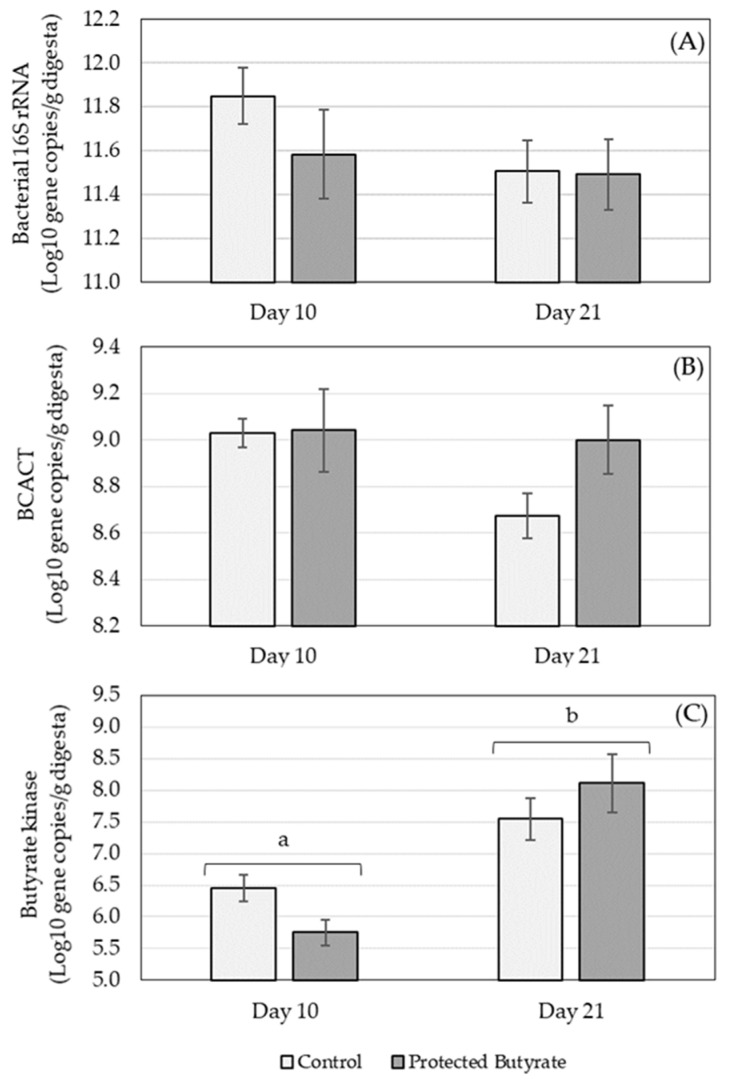
Concentration of gene copies in caecum ingesta of (**A**) bacterial 16S rRNA, (**B**) butyryl-CoA acetate CoA transferase (BCACT) and (**C**) butyrate kinase on different days of age (10 and 21) in broilers treated with protected butyrate compared to the control group. Bars represent the group means and error bars the standard error of the mean. Bars labelled with different letters are significantly different in terms of age (*p* ≤ 0.001).

The concentration of total eubacteria (based on the bacterial 16S rRNA gene) as well as key genes (butyrate kinase and BCACT) associated with the two different pathways for microbial production of butyrate was determined (Figure 1).

The concentration of bacterial 16S rRNA genes was not affected by treatment (*p* = 0.338), age (*p* = 0.183) or age × treatment (*p* = 0.443) (Figure 1A). Similarly, the concentration of BCACT was also not influenced by treatment (*p* = 0.196), age (*p* = 0.138) or age × treatment (*p* = 0.232) (Figure 1B). Butyrate kinase was significantly affected by age (*p* ≤ 0.001) with lower gene concentrations on day 10 compared to day 21 (Figure 1C). There was no significant treatment (*p* = 0.836) or age × treatment (*p* = 0.056) effect on butyrate kinase.

## 4. Discussion

The overall aim of this study was to develop new insights into the effects of orally supplemented protected butyrate on the poultry gut. A broiler performance study was first conducted, which informed the dosage level of the protected butyrate for the gut mechanistic study. As the mechanistic study used birds up to 21 days of age, the performance study focused on assessing different protected butyrate dosages during the starter phase, followed by a lower dosage in the grower phase. Lower feed dosage of protected butyrate in the grower phase is common practice in the feed industry due to the higher feed intake of older birds.

Based on its various biological actions, butyrate is known to improve the growth performance of broiler chickens, reflected by increased body weight gain and decreased FCR values [9]. It was also reported that microencapsulated butyrate and butyric acid glycerides increased carcass weight and breast meat yield of broilers [23,24], whose effects became more pronounced under suboptimal health conditions, such as Eimeria oocyst or Escherichia coli lipopolysaccharide (LPS) challenge [7,24]. In the performance study reported here, recycled broiler house litter was used, which is common practice in the broiler industry to lower production costs. This practice can also result in mild stressors, which, under experiment conditions, can more realistically simulate commercial conditions under which subclinical or mild clinical heath issues may occur [25]. As such, the results reported here should bear this in mind when comparing to studies performed with no recycled litter. However, previous studies both with [24] and without [23] challenges have reported improvements in performance as a result of butyrate supplementation.

In line with literature data, regardless of the dosage level used during the starter phase, protected butyrate significantly improved bird performance in the present study. This was due to an improved feed conversion ratio and increased average daily gain, without changing feed intake. It is clear from the findings though that the extent of the improvement was influenced by the starter phase dosage levels, as well as the production stage assessed. For example, the high dosage level (1.0 g/kg feed) significantly improved the body weight gain at the end of the starter phase, while the low dosage level (0.3 g/kg feed) did not, but, at the end of the grower phase, the approx. 70 g increase in body weight for both dosages only numerically differed from the control. In contrast, only the medium dosage level (0.6 g/kg feed) resulted in a significantly increased body weight at 35 days. Despite this, the high dosage level gave consistently the lowest feed conversion ratio which has clear value in terms of improving the sustainability of broiler production through the optimization of feed digestion efficiency. As such, the high dosage level (1 g/kg feed) was selected for use in the subsequent experiment to study the effects of protected butyrate within the gut. The applied doses are consistent with those of previous studies, ranging mostly from 0.25 to 1.0 g/kg unprotected sodium butyrate [7] or 0.5 to 1.0 g/kg microencapsulated sodium butyrate [17,26].

An overview of the results obtained in the gut mechanism study is presented in Table 6. The abundance of the major tight junction proteins, claudin-1 and claudin-3, was shown to be affected by supplementation of protected butyrate. In the jejunum+ileum samples, the tight junction proteins were both increased on the 10th and 21st days of age in chickens receiving protected butyrate supplementation. These findings are consistent with the necrotic enteritis challenge broiler study of Song et al. [17], who reported that butyrate supplementation increased gene expression of various tight junction proteins in the jejunum of broilers ranging in age from 19–21 and 25–27 days (i.e., 3 or 9 days post infection in the study). This indicates that butyrate benefits gut barrier integrity even in healthy birds. However, tight junction proteins are not limited to the jejunum. Therefore, in this study, the effect of protected butyrate supplementation was also assessed at other gut sites that were not investigated in previous studies with chickens. Concerning other gut sections, the stimulatory role of butyrate on claudin-3 and occludin was confirmed in the colon of weaned piglets [27], and the butyrate-triggered rearrangement of colonic tight junction proteins can contribute to the protective role of butyrate in inflammatory bowel disease in humans [28]. However, no literature data are available regarding gut sites proximal to the jejunum for poultry.

Effects of protected butyrate supplementation on tight junction proteins in the gizzard and duodenum were different to that of the jejunum. The responses of claudin-1 differed by gut site, increasing in the gizzard and decreasing in the duodenum. Claudin-3 in contrast was only affected by protected butyrate in the duodenum, which was age dependent: decreasing in 10 day old birds and increasing in 21 day old birds. As such, it is clear that the effect of protected butyrate on tight junction protein in the avian upper intestine is complex. It was also observed in other species that butyrate could influence tight junction protein expression in a multifaceted way. For instance, the gene expression of occludin was stimulated in the duodenum, while claudin-1 was downregulated in the duodenum, jejunum and ileum of weaned pigs fed a pre-starter diet with sodium butyrate supplementation (0.96 g/kg feed) [29]. Similarly to the absence of protected butyrate-triggered modulation of claudins in the caecal mucosa in the present study, the expression of tight junction proteins was not influenced by orally applied butyrate in the porcine colon [29]. The complexity of butyrate’s action on gut barrier function is also reflected by the multiple underlying mechanisms. Butyrate is known to promote the interplay of the SP1 transcription factor and the claudin-1 promoter [16], contributing to increased protein abundance. Besides the stimulation of claudin expression, butyrate also accelerates the assembly of tight junctions via the calcium/calmodulin-dependent protein kinase β mediated activation of the AMP-activated protein kinase as reported on the Caco-2 cell line [30,31], which is also critical for gut barrier integrity. The different action of butyrate at various gut sections and time points might be related to site- and age-dependently altered cell signaling pathways or to the interaction of the dynamically changing inflammatory homeostasis and the microbiome, both affected by butyrate as a key regulator [29].

As butyrate can have effects beyond the gut, the main SCFA transporter (MCT- 1) was also assessed along the broiler gut. In the upper intestine, MCT-1 was affected by protected butyrate supplementation in the same manner as claudin-1, except for a treatment × age interaction in the duodenum. Unlike claudin-1, in the duodenum on day 10, MCT-1 was decreased to a greater extent than on day 21.

In contrast to the upper intestine, in the caecum, the effect of protected butyrate supplementation on tight junction proteins and the SCFA transporter was limited. Only an effect on MCT-1 occurred, which was age dependent: decreasing in 10 day old birds. This limited effect in the caecum is consistent with the digesta butyrate concentrations, which indicated that the amount of supplemented protected butyrate reaching beyond the small intestine was minimal, particularly in 21 day old birds where jejunum+ileum butyrate concentrations were identical in the control and protected butyrate supplemented animals.

In a previous study of our research group, both dietary unprotected sodium butyrate supplementation and wheat-based diet triggering enhanced large intestinal butyrate production significantly increased MCT-1 protein abundance in the ileum of 6-week-old broilers (unpublished data). These results are in line with the butyrate evoked intense upregulation of MCT-1 in human colonic epithelial cells [32]. Similarly, a positive correlation was found between the expression of several MCT genes (MCT-1, -2, -8, -9, -10 and -14) and the intestinal butyrate concentration in the duodenum of broilers, whilst butyrate reduced MCT-9 gene expression in the ileum, with remaining further MCT genes unaffected [33]. The site-dependency of the protected butyrate’s modulatory role on MCT-1 was also demonstrated in the present study as this SCFA transporter was upregulated by protected butyrate in the gizzard and jejunum+ileum, but its protein abundance decreased in the duodenum. It should be stressed that the expression of MCT-1 is regulated by several mechanisms on both transcriptional and post-transcriptional levels, and SCFAs together with lactate as its major ligands can either up- or downregulate MCT-1 abundance based on the actual quantitative pattern of different monocarboxylates as well as the responsiveness of the gut mucosa.

In contrast to the tissue proteins, protected butyrate supplementation influenced digesta SCFA in all gut sites including the caecum. However, considering the gradual release and subsequent absorption of protected butyrate throughout the small intestines, this is suggested to be not simply due to protected butyrate supplementation. As indicated in previous studies, orally supplemented protected butyrate products mostly provide butyrate release and absorption from the lower small intestines, but do not possess a direct increasing action on caecal butyrate concentrations [34]. Hence, the elevation of butyrate concentration in the caecum of protected butyrate supplemented chicks observed in the present study should be related to the stimulation of the hindgut microbiome. 

In the gizzard and duodenum, protected butyrate supplementation was associated with a decreased acetate concentration that was much greater in extent than the associated increase of butyrate. It can be suggested that acetate absorption was remarkably facilitated by the enhanced MCT-1 protein abundance in the gizzard, contributing to decreased acetate concentration in the ingesta of butyrate-treated birds. The similar acetate decrease in the duodenal ingesta may be considered as a downstream effect of the enhanced gastric acetate absorption. However, in the case of butyrate, the stimulated absorption may be overcompensated by the gradual butyrate release from the applied microencapsulated form. Effects were also seen on propionate concentrations, although these were more complex due to being affected by gut site and bird age. Conversely, in the caecum, all major SCFA were consistently increased by protected butyrate supplementation resulting in an approx. 20% increase in total SCFA. As butyrate produced by the caecal microflora is suggested to be a potent modulator of insulin signaling in broilers [14], contributing towards increased tissue insulin sensitivity, the stimulation of intestinal SCFA production by the applied protected butyrate might be an important driver beyond the growth promoting effect observed in the present performance study.

As the SCFA in the caecum is normally generated by the resident microbiota, it was hypothesized that the increase in caecal SCFA concentration by protected butyrate supplementation was due to stimulation of endogenous microbial production. However, digesta concentrations of total eubacteria and genes associated with butyrate production were not significantly affected by protected butyrate supplementation. Notwithstanding that the total eubacteria count (reflected by 16S rRNA levels) was not altered by protected butyrate in the present study, the overall composition of the caecal microflora of broilers could be effectively modulated by oral sodium butyrate supplementation at the age of 21 days in a previous study [4]. However, delivering more substrates to the hindgut microbes by fiber-rich wheat-based diet had a stronger stimulus on bacterial communities than orally supplied butyrate [4]. On this basis, it is speculated that the increased endogenous production of SCFA was linked to increased microbial activity. Future studies should consider the use of metagenomic and/or metatranscriptomic analysis of the caecal microbiota in order to confirm if this is indeed the case.

How might supplementation of protected butyrate result in increased activity of the caecal microbiota? From the performance study, it is clear that protected butyrate supplementation improved the feed conversion ratio indicating improved feed digestibility, as has been reported in other broiler studies [7,17,26]. The ability of butyrate supplementation to increase digestive efficiency has been linked to increased digestive secretions in the stomach and small intestine [35]. This is likely to alter the fermentable material reaching the large intestine, which could modify microbial activity. Such an effect is well known as supplementation of fiber-rich wheat-based broiler diets with non-starch polysaccharide-degrading enzymes (e.g., xylanase) can stimulate SCFA production by the hindgut microbiota [34,36]. Furthermore, butyrate absorbed from the small intestines can also serve as a key messenger molecule affecting several pathways in downstream gut sections as well [34] and serving as a link between the host and the microbiome [37]. 

## 5. Conclusions

Based on the findings of this study, the positive modulatory effects of protected butyrate on growth performance, claudin-1 and -3 tight junction protein and MCT-1 transporter abundance have been reported in various gastrointestinal segments. The beneficial effect of protected butyrate on the caecal microbiota was also successfully described by quantifying the concentration of total eubacteria and enzymes involved in two different pathways for microbial production of butyrate. Furthermore, an increase in caecal SCFA production as well as alterations in SCFA composition induced by oral protected butyrate supplementation has been demonstrated, which may be the result of increased microbial activity. In summary, use of protected butyrate as a feed additive has various beneficial effects on the gut microbiota as well as on the performance, overall health status and metabolism of the host organism as highlighted by the presented study findings. This information provides valuable insight into the complex effects of the biologically most active SCFA, butyrate, for poultry. Regarding future perspectives, further studies are necessary to better understand the effects of orally applied protected butyrate on the gut microbiota. This will generate insights into the underlying mechanisms by which butyrate can modulate the gut microbiome, endogenous SCFA production and metabolic health.

## Figures and Tables

**Table 1:** Details of the basal diets used for the broiler performance study.

Parameter	Starter (1–14 days)	Grower (15–35 days)
Ingredients (%):		
Corn	59.31	62.64
Soybean meal	34.36	29.66
Soybean oil	2.00	3.68
Sodium bicarbonate	0.15	0.15
Sodium chloride	0.21	0.21
Limestone	1.30	1.16
Monocalcium phosphate	1.82	1.64
Choline chloride	0.09	0.09
DL-Methionine	0.26	0.23
L-Lysine	0.16	0.19
L-Threonine	0.09	0.10
Vitamins & trace minerals	0.25	0.25
Calculated Nutritional Values:		
ME (kcal/kg)	2950	3100
Crude protein (g/kg)	215.0	195.0
Lysine (g/kg)	12.50	11.50
Methionine (g/kg)	5.86	5.34
Met + Cys (g/kg)	9.05	8.70
Threonine (g/kg)	9.50	8.30
Tryptophan (g/kg)	2.51	2.25
Calcium (g/kg)	10.00	9.00
Phosphorus (g/kg)	5.00	4.50

**Table 2:** Details of the basal diets used for the gut mechanism study.

Parameter	Starter (1–10 days)	Grower (11–21 days)
Ingredients (%):		
Corn	33.88	22.55
Wheat (incl. xylanase)	25.00	40.00
Soybean meal	26.00	24.00
Rapeseed meal	4.00	5.00
Full fat soyabeans	5.00	-
Animal fat	-	4.00
Soyabean oil	2.25	1.20
Limestone	1.75	1.40
Monocalcium phosphate	0.70	0.40
Vitamins & trace minerals	0.50	0.50
Sodium bicarbonate	0.24	0.21
Sodium chloride	0.22	0.21
DL-Methionine	0.22	0.20
L-Lysine HCl	0.17	0.22
L-Threonine	0.05	0.07
L-Valine	0.01	0.03
Phytase	0.01	0.01
Calculated Nutritional Values:		
ME (kcal/kg)	2849	2963
Crude protein (g/kg)	215	201
Lysine (g/kg)	12.5	11.7
Methionine (g/kg)	5.5	5.1
Met + Cys (g/kg)	9.2	8.6
Threonine (g/kg)	8.4	8.0
Tryptophan (g/kg)	2.6	2.4
Calcium (g/kg)	9.0	7.2
Phosphorus (g/kg)	5.6	4.8

**Table 3:** Effect of different dosages of protected butyrate on broiler performance ^#^.

Parameter	Control	Low	Medium	High	Pooled SEM	*p*	*p* Linear	*p* Square
Body Weight (g):								
Day 1	37.4	37.5	37.4	37.4	0.028	0.607	-	-
Day 14	350.8 ^b^	361.3 ^ab^	368.3 ^a^	369.4 ^a^	2.479	0.024	0.042	0.210
Day 35	1960.3 ^b^	2030.4 ^b^	2113.2 ^a^	2029.3 ^b^	14.722	0.001	<0.001	0.002
Average Daily Gain (g):								
Days 1–14	22.4 ^b^	23.1 ^ab^	23.6 ^a^	23.7 ^a^	0.177	0.023	0.042	0.210
Days 15–35	76.6 ^b^	79.6 ^ab^	83.0 ^a^	79.1 ^ab^	0.626	0.002	<0.001	0.001
Days 1–35	54.9 ^c^	57.5 ^ab^	59.3 ^a^	56.9 ^bc^	0.431	0.002	<0.001	0.001
Average Daily Feed Intake (g):								
Days 1–14	33.0	32.7	32.9	32.7	0.230	0.942	0.876	0.957
Days 15–35	121.6	125.1	125.9	121.4	0.828	0.114	0.022	0.015
Days 1–35	86.2	88.1	88.7	85.5	0.517	0.090	0.023	0.013
Feed Conversion Ratio:								
Days 1–14	1.460	1.401	1.394	1.391	0.012	0.132	0.075	0.202
Days 15–35	1.579 ^b^	1.551 ^ab^	1.534 ^a^	1.518 ^a^	0.003	<0.001	0.024	0.339
Days 1–35	1.559 ^c^	1.525 ^b^	1.511 ^ab^	1.496 ^a^	0.006	<0.001	0.006	0.170
Livability (%)	97.8	100.0	100.0	96.7	0.588	0.106	-	-

^#^ Values in same row with no common superscript (a, b, c) are significantly different (*p* ≤ 0.05).

**Table 4:** Effect of protected butyrate supplementation on tight junction proteins (claudin-1 and claudin-3) and a SCFA transporter (MCT-1) in the broiler gut at different days of age.

Parameter (ng/mg Protein)	Day 10		Day 21		Pooled SEM	*p*-Value		
	Control	Protected Butyrate	Control	Protected Butyrate		Age	Treatment	Interaction
Gizzard:								
Claudin-1	0.307	0.316	0.111	0.154	0.010	<0.001	0.010	0.160
Claudin-3	0.871	0.578	0.278	0.359	0.047	<0.001	0.106	0.002
MCT-1	0.625	0.723	0.280	0.409	0.019	<0.001	<0.001	0.780
Duodenum:								
Claudin-1	0.248	0.223	0.117	0.091	0.012	<0.001	0.041	0.968
Claudin-3	0.428	0.347	0.189	0.189	0.022	<0.001	0.078	0.079
MCT-1	0.516	0.377	0.128	0.119	0.026	<0.001	0.006	0.015
Jejunum + Ileum:								
Claudin-1	0.240	0.364	0.128	0.157	0.025	<0.001	0.004	0.061
Claudin-3	0.528	0.609	0.280	0.388	0.028	<0.001	0.002	0.640
MCT-1	0.697	0.881	0.172	0.374	0.041	<0.001	<0.001	0.823
Caecum:								
Claudin-1	0.435	0.387	0.184	0.192	0.022	<0.001	0.376	0.211
Claudin-3	1.197	0.990	0.460	0.480	0.060	<0.001	0.127	0.064
MCT-1	0.876	0.661	0.339	0.349	0.052	<0.001	0.053	0.041

**Table 5:** Effect of protected butyrate supplementation on individual and total SCFA concentrations in the broiler gut at different days of age.

Gut Site	Parameter (μmol/g Digesta)	Day 10		Day 21		Pooled SEM	*p*-Value		
		Control	Protected Butyrate	Control	Protected Butyrate		Age	Treatment	Interaction
Gizzard	Acetate	6.72	5.24	5.48	4.79	0.387	0.053	0.012	0.348
	Propionate	0.272	0.384	0.305	0.262	0.023	0.053	0.130	0.002
	Butyrate	0.036	0.196	0.029	0.184	0.020	0.678	<0.001	0.918
	Valerate	0.012	0.009	0.009	0.008	0.002	0.158	0.116	0.435
	Total SCFA	7.04	5.82	5.83	5.24	0.402	0.049	0.044	0.472
Duodenum	Acetate	3.57	3.02	6.04	3.14	0.364	0.001	<0.001	0.004
	Propionate	0.206	0.314	0.425	0.204	0.023	0.026	0.023	<0.001
	Butyrate	0.023	0.039	0.021	0.037	0.004	0.688	0.001	0.955
	Valerate	0.012	0.009	0.010	0.008	0.002	0.493	0.222	0.980
	Total SCFA	3.81	3.38	6.50	3.39	0.376	0.001	<0.001	0.001
Jejunum + Ileum	Acetate	6.51	5.85	3.88	4.60	0.390	<0.001	0.980	0.089
	Propionate	0.218	0.248	0.181	0.156	0.013	<0.001	0.809	0.043
	Butyrate	0.011	0.016	0.009	0.009	0.001	<0.001	0.005	0.002
	Valerate	0.010	0.008	0.005	0.005	0.001	0.158	0.048	0.434
	Total SCFA	6.75	6.12	4.08	4.77	0.396	<0.001	0.974	0.107
Caecum	Acetate	79.37	90.51	62.66	75.37	4.877	0.003	0.023	0.877
	Propionate	2.18	2.85	3.43	4.53	0.421	0.002	0.047	0.623
	Butyrate	5.83	8.36	7.44	10.64	1.044	0.079	0.012	0.760
	Valerate	0.055	0.063	0.514	0.817	0.097	<0.001	0.105	0.148
	Total SCFA	87.44	102.10	74.05	91.36	5.604	0.046	0.009	0.820

**Table 6:** Overview of the significant (*p* < 0.05) effects of age and dietary treatment obtained in the gut mechanism study. Upstream arrows (↑) refer to significantly increased, while downstream arrows (↓) indicate significantly declined means of day 10 vs. day 21 or butyrate supplemented vs. control animals.

Gut Site	Parameter	Age	Butyrate
Gizzard	Claudin-1	↓	↑
	Claudin-3	↓	-
	MCT-1	↓	↑
	Acetate	↓	-
	Propionate	-	-
	Butyrate	-	↑
	Valerate	-	-
	Total SCFA	↓	↓
Duodenum	Claudin-1	↓	↓
	Claudin-3	↓	-
	MCT-1	↓	↓
	Acetate	↑	↓
	Propionate	↑	↓
	Butyrate	-	↑
	Valerate	-	-
	Total SCFA	↑	↓
Jejunum + Ileum	Claudin-1	↓	↑
	Claudin-3	↓	↑
	MCT-1	↓	↑
	Acetate	↓	-
	Propionate	↓	-
	Butyrate	↓	↑
	Valerate	-	↓
	Total SCFA	↓	-
Caecum	Claudin-1	↓	-
	Claudin-3	↓	-
	MCT-1	↓	-
	Acetate	↓	↑
	Propionate	↑	↑
	Butyrate	↑	↑
	Valerate	↑	↑
	Total SCFA	↓	↑
	Bacterial 16S RNA	-	-
	BCACT	-	-
	Butyrate kinase	↑	-

## Data Availability

The datasets generated during and/or analyzed during the current studies are available from the corresponding author on reasonable request.
